# Vision Screening at School: What Do Primary Teachers Know, and How Can They Help?

**DOI:** 10.22599/bioj.495

**Published:** 2025-12-05

**Authors:** Chaimae El Harrak, Mustapha Jaouhari, Farida Bentayeb, Youssef Elmerabet

**Affiliations:** 1 Laboratory of Electronic, Mechanical, and Energetic Information Processing Systems, Faculty of Sciences, Ibn Tofail University, Morocco; 2 Laboratory of Engineering and Materials (LIMAT), Hassan II University of Casablanca, Faculty of Sciences Ben M’Sick, Morocco

**Keywords:** Refractive error, children, school, screening, strabismus, education

## Abstract

**Background:**

Vision plays a critical role in a child’s educational success, cognitive development, and social interaction. However, many children, particularly in low- and middle-income countries like Morocco, suffer from undiagnosed visual impairments during their school years. As teachers spend extended time with students, they are well-positioned to notice early signs of vision problems.

**Objectives:**

This study aimed to assess the level of knowledge that Moroccan primary school teachers have about children’s visual health, identify gaps in awareness of common visual disorders, explore teachers’ roles in detecting signs of visual issues in the classroom, and support the development of school-based vision screening strategies.

**Methods:**

A cross-sectional descriptive study was conducted among 271 primary school teachers across different Moroccan regions. Data were collected using a structured questionnaire covering demographic characteristics, knowledge of common visual disorders, awareness of behavioural signs, and current classroom practices. Statistical analyses were performed to examine associations between knowledge levels and variables such as gender, age, and teaching experience.

**Results:**

The majority of participants (84.9%) reported more than five years of teaching experience, corresponding to an estimated average of approximately nine years in the profession. Teachers demonstrated a better understanding of refractive errors and strabismus compared to ocular surface diseases and behavioural signs. Teaching experience was significantly associated with higher knowledge scores (p = 0.001), whereas no significant differences were found based on gender. Although age was not directly correlated with knowledge scores, older teachers were significantly more likely to report observing academic improvement in students after correcting visual problems (p = 0.0007), a perception also more frequent among those with greater teaching experience (p = 0.004). Despite existing knowledge gaps, teachers reported supportive practices such as adjusting classroom seating (94.5%) and informing parents (52.6%).

**Conclusion:**

The findings highlight the potential value of providing targeted training programmes to help teachers play a more active role in recognising visual problems in children. Incorporating basic eye health education into teacher training curricula may support earlier detection and contribute to better child health outcomes in school environments, though further research is recommended to validate this approach.

## Introduction

Visual health is a key aspect of a child’s overall well-being, directly impacting cognitive development, school performance, and social integration (Gilbert and Foster, 2001). Globally, uncorrected visual impairments such as refractive errors, amblyopia, and congenital anomalies continue to be major causes of avoidable vision loss in children (Rahi and Gilbert, 2012). In low- and middle-income countries, including Morocco, access to routine paediatric eye care remains limited, and many children live with undetected visual problems throughout critical years of learning.

In the Moroccan context, this issue is particularly concerning. While the national education system has made significant strides towards promoting inclusive education, there is currently no structured school-based visual screening programme implemented on a large scale in public primary schools. Most vision problems are only identified after they begin to significantly impact classroom performance (Basch, 2011). Unfortunately, by the time intervention occurs, valuable time may have already been lost in terms of academic progress and psychosocial development.

Teachers are often the first adults, aside from family members, to observe signs that may indicate a child has visual difficulties, such as squinting at the board, getting too close to books, or complaining of headaches (Habiba *et al*., 2017). Yet in Morocco, as in many developing nations, teachers typically receive no formal training on how to recognise or respond to such signs. This gap may impede early detection efforts, especially in rural and underserved areas where access to ophthalmologists or optometrists is even more limited.

Studies in countries such as Pakistan (Habiba *et al.*, 2017) and Swaziland (Sukati, Mashige and Moodley, 2021) have demonstrated that teachers’ knowledge, attitudes, and practices toward children’s visual health range from low to moderate. These findings suggest that a similar knowledge gap could exist among Moroccan teachers, although few local studies have explored this issue.

Moreover, integrating teachers into a coordinated school health strategy has shown promising results. Studies from India have demonstrated that training teachers to conduct basic vision screenings can effectively support early identification and referral of children with visual impairments, even in resource-limited settings (Sudhan *et al*., 2024). This model could be adapted in Morocco, where teachers already play a key role in children’s overall development and are trusted figures within their communities.

The present study seeks to assess the level of knowledge that primary school teachers in Morocco have regarding children’s visual health. It also aims to explore their readiness and perceived role in the early detection of visual problems in the classroom. The insights gained may help inform future training programmes and collaborative school health policies that bridge the gap between education and eye care services in Morocco.

## Methods and Materials

This was a cross-sectional, descriptive study conducted using a structured questionnaire designed to evaluate primary school teachers’ knowledge, perceptions, and practices regarding children’s visual health. The study targeted primary school teachers from various regions of Morocco, representing a diverse range of educational settings. An official authorisation was obtained from the Moroccan Ministry of National Education to carry out this study.

In collaboration with educational administrators, the questionnaire was distributed via verified WhatsApp and Gmail groups exclusively dedicated to primary school teachers. This ensured that only eligible participants had access to the survey. A non-probability convenience sampling technique was employed to reach teachers from different cities and provinces across Morocco.

The self-administered questionnaire was designed to explore teachers’ knowledge and attitudes related to children’s visual health. It was composed of four main parts:

### Part 1 – Demographic information: Q1–Q4

This section gathered basic information, including gender, age, years of teaching experience and grade level currently taught.

### Part 2 – Knowledge assessment (scored section): Q5–Q8

This part included multiple-choice questions assessing the participants’ knowledge of visual health. Each question had several correct answers, and the scoring system was as follows: all correct answers selected: 3 points (Good knowledge), three correct answers: 2 points (Moderate knowledge), and two or fewer correct answers: 1 point (Low knowledge).

The topics in this section included types of refractive errors known by the teacher, the definition of strabismus, the identification of ocular surface diseases, and behavioural or academic signs that may indicate a visual disorder in a student.

### Part 3 – Yes/no questions (attitudes and perceptions): Q9–Q11

This section consisted of closed-ended questions (Yes/No) assessing participants’ awareness, experience, and attitudes, such as: Have you ever observed academic improvement in a student after correcting a visual problem (e.g., wearing glasses)? Have you noticed that visual problems affect a child’s psychological state, classroom participation, and interaction? Have you ever participated in a training that included a component on visual health?

### Part 4 – Reflection and practise-oriented questions: Q12–Q13

This final section explored how teachers respond to visual health issues in practice and their interest in professional development. It included questions such as: How do you respond if a child in your class has a visual problem? Would you be willing to participate in a workshop or training on visual health?

To ensure accessibility and clarity, the questionnaire was made available in both Arabic and French. Participation in the study was entirely voluntary and anonymous. No identifying personal information was collected, and confidentiality of all responses was strictly maintained.

Data were analysed using both descriptive and inferential statistical methods. Descriptive statistics (frequencies, percentages, and means) were used to summarise the participants’ demographic characteristics and knowledge levels. For inferential analysis, the Kruskal-Wallis test was employed to assess differences in knowledge scores across multiple demographic groups (e.g., age categories, teaching experience), while chi-square tests were used to examine associations between categorical variables. To control for the increased risk of Type I errors due to multiple comparisons, a Bonferroni correction was applied. Statistical significance was set at a p-value of less than 0.05.

## Results

A total of 271 primary school teachers from different regions of Morocco completed the questionnaire. As detailed in Table 1, the sample included 163 women (60.1%) and 108 men (39.9%). Most participants were experienced educators, with 84.9% reporting more than five years of teaching experience. In terms of age distribution, the majority were aged between 35 and 45 years (39.5%), followed by those over 45 years (33.9%). Teachers under 25 years of age represented only a small portion of the sample (3.7%).

**Table 1:** Characteristics of the participating primary school teachers (N = 271), including gender, age group, and years of teaching experience.


CATEGORY	SUBCATEGORY	NUMBER OF PARTICIPANTS

**Gender**	Women	163

	Men	108

**Age Group**	Under 25 years	10

	Between 25 and 35 years	62

	Between 35 and 45 years	107

	Over 45 years	92

**Teaching Experience**	Less than 5 years	41

	More than 5 years	230

	Total	271


The results of the knowledge-based questions (Tables 2, 3, 4 and 5) revealed varying levels of understanding about children’s visual health. Teachers were categorised into three groups—good, medium, and low—based on their responses to multiple-choice items. While there were no statistically significant differences in knowledge scores by gender (p = 0.17) or age (p = 0.36), teaching experience was a determining factor. Specifically, teachers with more than five years of experience demonstrated significantly higher scores regarding their ability to identify common visual conditions such as refractive errors and strabismus (p = 0.001). Although overall knowledge of strabismus and ocular surface diseases remained low across the sample, a near-significant trend was observed for strabismus knowledge and years of teaching (p = 0.07), suggesting that increased classroom exposure may improve familiarity with such conditions.

**Table 2:** Q5. Types of refractive errors known by the teacher.


VARIABLE	CATEGORY	GOOD	MEDIUM	LOW	p-VALUE (BONFERRONI CORRECTED)

**Gender**	Female	75	74	14	0.17¹

	Male	38	61	9	

**Age Group**	Under 25	6	2	2	0.36^2^

	25–35	22	30	9	

	35–45	49	53	6	

	Over 45	36	50	6	

**Experience**	<5 years	5	10	12	**0.0001¹**

	>5 years	108	125	11	


^1^Chi-square test. ^2^Fisher’s exact test.

**Table 3:** Q6. Knowledge of the definition of strabismus.


VARIABLE	CATEGORY	GOOD	MEDIUM	LOW	p-VALUE (BONFERRONI CORRECTED)

Gender	Female	18	16	129	0.17¹

	Male	10	3	95	

Age Group	Under 25	0	1	9	0.83^2^

	25–35	6	4	51	

	35–45	12	9	87	

	Over 45	10	5	77	

Experience	<5 years	1	4	35	0.07¹

	>5 years	27	15	189	


^1^Chi-square test. ^2^Fisher’s exact test.

**Table 4:** Q7. Identification of ocular surface diseases.


VARIABLE	CATEGORY	GOOD	MEDIUM	LOW	p-VALUE (BONFERRONI CORRECTED)

Gender	Female	20	10	133	0.19¹

	Male	17	12	79	

Age Group	Under 25	3	0	7	0.85^2^

	25–35	4	9	48	

	35–45	18	5	85	

	Over 45	12	8	72	

Experience	<5 years	3	5	32	0.38¹

	>5 years	34	17	180	


^1^Chi-square test. ^2^Fisher’s exact test.

**Table 5:** Q8. Behavioural or academic signs suggesting a visual disorder in a student.


VARIABLE	CATEGORY	GOOD	MEDIUM	LOW	p-VALUE (BONFERRONI CORRECTED)

Gender	Female	26	18	119	0.19¹

	Male	20	17	71	

Age Group	Under 25	5	0	12	0.85^2^

	25–35	6	7	48	

	35–45	18	8	80	

	Over 45	10	5	72	

Experience	<5 years	9	9	22	0.38¹

	>5 years	34	17	180	


^1^Chi-square test. ^2^Fisher’s exact test. **Bold** values indicate statistically significant p-values (p < 0.05).

Regarding the identification of behavioural or academic signs that could indicate visual problems, knowledge levels were again predominantly low, with no significant associations found with gender (p = 0.19), age (p = 0.85), or experience (p = 0.38). As shown in Table 6, teacher’ engagement with health-related programmes was limited. Only 43 teachers had previously participated in general child health training, and just 15 had attended awareness sessions specifically focused on visual health. Despite this, a large majority (94.5%) believed that correcting a visual problem leads to academic improvement (Table 7). This belief was significantly more common among older teachers (p = 0.0007) and those with more than five years of experience (p = 0.0038), suggesting a greater awareness of the impact of vision on learning within these groups.

**Table 6:** Q11. Teacher participation in visual health awareness programmes.


VARIABLE	CATEGORY	YES	NO	p-VALUE (BONFERRONI CORRECTED)

Gender	Female	7	61	0.74¹

	Male	8	50	

Age Group	Under 25	0	6	–

	25–35	4	24	0.77^2^

	35–45	7	47	0.98^2^

	Over 45	4	34	

Experience	<5 years	1	16	0.67¹

	>5 years	14	95	


**Table 7:** Q9. Perceived academic improvement in a student after correcting a visual problem.


VARIABLE	CATEGORY	YES	NO	p-VALUE (BONFERRONI CORRECTED)

Gender	Female	155	3	0.58¹

	Male	101	4	

Age Group	Under 25	9	1	**0.0007^2^**

	25–35	49	5	

	35–45	107	0	

	Over 45	91	1	

Experience	<5 years	31	4	**0.0038¹**

	>5 years	225	3	


Psychological awareness, shown in Table 8, was also high, with 88.2% of respondents acknowledging that visual difficulties may affect children’s emotional well-being and classroom participation. This awareness was significantly associated with teaching experience (p = 0.03), but not with gender (p = 0.06) or age (p = 0.17). However, participation in visual health programmes remained low across all demographic categories, with no significant differences observed by gender (p = 0.74), age (p = 0.98), or experience (p = 0.67). Encouragingly, an overwhelming 85.9% of teachers expressed willingness to attend future workshops or educational sessions on visual health, indicating a clear interest in continuing professional development in this area.

**Table 8:** Q10. Teacher awareness of the psychological effects of visual difficulties.


VARIABLE	CATEGORY	YES	NO	p-VALUE (BONFERRONI CORRECTED)

Gender	Female	139	24	0.06¹

	Male	101	7	

Age Group	Under 25	9	1	0.77^2^

	25–35	49	12	

	35–45	95	13	0.17^2^

	Over 45	87	5	

Experience	<5 years	31	9	**0.035¹**

	>5 years	209	22	


^1^Chi-square test. ^2^Fisher’s exact test. **Bold** values indicate statistically significant p-values (p < 0.05).

**Figure 1:**
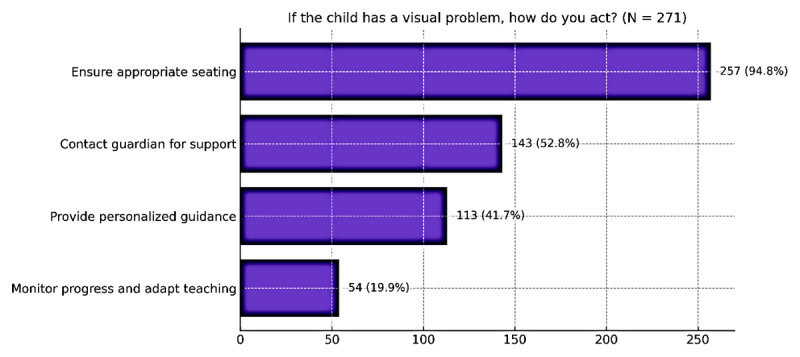
Teachers’ responses when a student has a visual problem (N = 271).

Teachers were also asked about their responses when encountering a student with suspected visual problems (Figure 1). The most common strategy, reported by 94.8% of participants, was ensuring that the student was seated in an appropriate place within the classroom. Additionally, 52.8% reported contacting the student’s guardian to discuss support strategies, while 41.7% provided personalised assistance during lessons. Notably, 19.9% stated that they actively monitored the student’s progress over time. These findings suggest that although formal training remains rare, many teachers intuitively implement inclusive and supportive practises, underscoring the importance of structured training programmes to reinforce and formalise these efforts.

## Discussion

This study sheds light on the critical role that Moroccan primary school teachers can play in the early detection of visual disorders, while also revealing important knowledge gaps that must be addressed to improve children’s eye health outcomes in school settings.

Despite the fact that 84.9% of the surveyed teachers had more than five years of experience, their overall knowledge of visual health, particularly regarding strabismus, ocular surface diseases, and behavioural signs, was moderate to low. These findings are consistent with studies conducted in other low- and middle-income countries. For example, Sukati *et al*. (2021) in Swaziland found that although teachers often showed concern for their students’ well-being, their knowledge of specific eye conditions was limited due to the absence of formal training. Similar trends were reported by Habiba *et al*. (2017) in Pakistan, emphasising the global nature of this issue.

One of the most striking findings of our study is the significant association between teaching experience and higher knowledge scores, particularly regarding refractive errors. This suggests that experiential learning exposure for students who demonstrate visible visual difficulties may help teachers identify certain conditions more effectively. For strabismus, while the association with experience was not statistically significant, a near-significant trend was observed, indicating that increased classroom exposure may gradually enhance familiarity with this condition. These findings are supported by Okasheh-Otoom *et al*. (2023), who found that teaching experience, combined with basic training, significantly enhanced the ability of schoolteachers in Jordan to accurately screen for refractive errors and other ocular abnormalities.

In contrast, variables such as gender and age were not significantly associated with knowledge levels. This finding may point to a systemic gap in pre-service training, which appears to be uniformly insufficient across demographics. As highlighted by Wedner *et al*. (2000), even highly motivated and observant teachers require structured training programmes to accurately identify visual impairments.

Although formal participation in visual health programmes was limited (only 15 teachers reported attending such training), many teachers demonstrated strong intuitive practices. For instance, 94.8% of participants reported adjusting classroom seating for children with suspected visual problems. This is encouraging, as previous research has emphasised the importance of environmental adjustments for visually impaired students. According to the American Optometric Association (2019), simple actions like optimal seating positions and reducing glare can significantly enhance visual access and learning for affected children.

Teachers’ perceptions regarding the academic and psychological effects of visual impairments were also notably high. A majority (94.5%) believed that correcting a visual issue could improve academic performance, and 88.2% acknowledged its psychological impact. These findings are corroborated by Basch (2011), who noted that untreated visual disorders are associated with poor academic performance, reduced self-esteem, and behavioural problems. Interestingly, this awareness was particularly pronounced among older and more experienced teachers, perhaps reflecting their greater exposure to long-term student outcomes.

However, the persistently low recognition of ocular surface diseases and behavioural indicators, despite high awareness of their effects, suggests a disconnect between theoretical understanding and practical identification. As Gilbert and Foster (2001) argue, even common signs such as eye rubbing, frequent blinking, or avoidance of near tasks are often missed without targeted training. This further justifies integrating basic ophthalmological concepts into teacher education curricula.

The willingness of 85.9% of participants to engage in future training is a promising finding. It reflects both the professional responsibility that teachers feel and the openness to cross-sector collaboration. In a study by Reddy *et al*. (2015), schools that integrated vision screening into their curriculum with teacher support achieved significantly higher referral rates and follow-up care for students compared to those relying solely on periodic visits by eye care professionals.

Given the scarcity of ophthalmologists and optometrists in many Moroccan regions, especially in rural areas, teachers may serve as the first line of detection in a tiered vision care model. This is supported by Burnett *et al*. (2018), who demonstrated through a systematic review that school-based eye care interventions led by trained teachers are both effective and scalable in low- and middle-income countries, especially when integrated with referral systems and supported by periodic retraining.

This study has several limitations. First, the use of a convenience sampling method may limit the generalisability of the findings across all Moroccan schools. Second, although the questionnaire was designed to assess knowledge and perceptions, the phrasing of certain questions, particularly those referring to clinical terms like ‘strabismus’ and ‘ocular surface diseases’, may have affected comprehension. These terms might not be familiar to many teachers, especially in the absence of prior medical training. Using simpler, lay terminology could have yielded more accurate reflections of participants’ knowledge and beliefs. Future versions of the questionnaire should consider this to enhance clarity and validity. Additionally, the self-reported nature of the data introduces the possibility of social desirability bias, and the lack of a follow-up clinical evaluation limits conclusions about actual screening effectiveness.

## Conclusion

This study highlights the pivotal role that Moroccan primary school teachers can play in the early detection of children’s visual problems. While most participants demonstrated a general awareness of the importance of visual health, their detailed knowledge, particularly concerning strabismus, ocular surface diseases, and behavioural indicators, remained limited. Teaching experience emerged as a significant factor influencing knowledge levels, suggesting that practical exposure contributes to improved awareness. Despite the lack of formal training, many teachers reported adaptive classroom practices such as adjusting seating arrangements and communicating with parents, reflecting an intuitive understanding of how vision affects learning. These findings emphasise the need for structured educational initiatives that equip teachers with the necessary skills to recognise visual disorders accurately. Integrating basic eye health education into teacher training programmes and promoting collaboration between the education and health sectors could strengthen early detection and referral systems in Moroccan schools. Such measures would not only improve children’s academic performance and psychosocial well-being but also contribute to broader public health goals of reducing avoidable visual impairment. Future research should evaluate the effectiveness of targeted training modules and assess their long-term impact on student outcomes and community eye health.

## Appendix

### Questionnaire

**Part 1 – Demographic information (Q1–Q5)**

1. **Age**
  - Under 25 years
  - Between 25 and 35 years
  - Between 35 and 45 years
  - Over 45 years
2. **Gender**
  - Male
  - Female
3. **Years of teaching experience**
  - Less than 5 years
  - More than 5 years
4. **Grade level you teach**
  - First grade
  - Second grade
  - Third grade
  - Fourth grade
  - Fifth grade
  - Sixth grade
5. **Type of institution**
  - Public
  - Private

**Part 2 – Knowledge assessment (Q6–Q9)**

(Questions not scored individually but used for global knowledge score)

6. **What are the signs that may indicate a visual disorder in a student?**
  *(Multiple answers allowed)*
  - Brings book or board too close to eyes
  - Squints to see better
  - Frequently rubs eyes
  - Avoids reading
  - Tilts head while reading
  - I don’t know
7. **Based on your experience, what are the most common visual problems you have observed in children?**
  - Strabismus
  - Reduced vision
  - Eye redness
  - Other
8. **Have you ever received training in child health?**
  - Yes
  - No
9. **If yes, did the training include visual health?**
  - Yes
  - No

**Part 3 – Attitudes and perceptions (Q10–Q11)**

10. **Have you noticed that visual problems affect the child’s psychological well-being, participation, and interaction in class?**
  - Yes
  - No
11. **Have you ever noticed academic improvement in a student after treating a visual issue (e.g., wearing glasses)?**
  - Yes, a significant improvement
  - Yes, a slight improvement
  - No improvement

**Part 4 – Practise-oriented questions (Q12–Q13)**

12. **If a child has a visual problem, how do you respond?**
  - I provide additional support and guidance in class
  - I make sure the student sits in a comfortable and suitable place
  - I discuss with the parents to offer necessary support
  - I monitor the case regularly and adapt teaching strategies as needed
13. **Would you be interested in attending a workshop or training session on visual health?**
  - Yes
  - No
